# Myeloid-Derived Suppressor Cells Are Increased in Lung Transplant Recipients and Regulated by Immunosuppressive Therapy

**DOI:** 10.3389/fimmu.2021.788851

**Published:** 2022-01-10

**Authors:** María Iglesias-Escudero, David San Segundo, David Merino-Fernandez, Victor M. Mora-Cuesta, Patricia Lamadrid, Marta Alonso-Peña, Sandra Raso, David Iturbe, Sonia Fernandez-Rozas, Jose Cifrian, Marcos López-Hoyos

**Affiliations:** ^1^ Transplant and Autoimmunity group, Research Institute-IDIVAL, Santander, Spain; ^2^ Immunology Department, Universitary Hospital Germans Trias i Pujol, Badalona, Spain; ^3^ Immunology Department, Universitary Hospital Marqués de Valdecilla-IDIVAL, Santander, Spain; ^4^ Pneumology Department, Universitary Hospital Marqués de Valdecilla-IDIVAL, Santander, Spain; ^5^ Molecular Biology Department, Universidad Cantabria, Santander, Spain

**Keywords:** lung transplantation, immunosuppression, rejection, myeloid derived suppressor cells, tacrolimus

## Abstract

Lung transplantation remains as a primary treatment for end-stage lung diseases. Although remarkable improvement has been achieved due to the immunosuppressive protocols, long-term survival for lung transplant recipients (LTR) is still limited. In the last few decades, an increasing interest has grown in the study of dysregulation of immune mechanisms underlying allograft failure. In this regard, myeloid-derived suppressor cells (MDSCs) could play an important role in the promotion of graft tolerance due to their immune regulatory function. Here, we describe for the first time circulating subsets MDSCs from LTR at several time points and we evaluate the relationship of MDSCs with sort-term lung transplant outcomes. Although no effect of MDSCs subsets on short-term clinical events was observed, our results determine that Mo-MDSCs frequencies are increased after acute cellular rejection (ACR), suggesting a possible role for Mo-MDSCs in the development of chronic lung allograft dysfunction (CLAD). Therefore, whether MDSCs subsets play a role as biomarkers of chronic rejection remains unknown and requires further investigations. Also, the effects of the different immunosuppressive treatments on these subpopulations remain under research and further studies are needed to establish to what extend MDSCs immune modulation could be responsible for allograft acceptance.

## Introduction

Lung transplantation remains the primary treatment option for patients with end stage lung failure. Despite the advance in the handling of lung transplant recipients, in contrast to heart, liver and kidney transplantation, 5-year survival in lung transplant recipients (LTR) remains limited (1) by post transplant development of bronchiolitis obliterans syndrome (BOS) which is the prime source of chronic allograft failure (2, 3).

Due to this, predicting the risk of developing chronic lung rejection is one of the most important aims in lung transplantation. However, the current understanding of the unbalanced regulatory mechanisms underlying chronic lung rejection is incomplete and identifying potential prognostic markers is necessary to achieve this aim.

In the last few years, there has been an increasing interest in the field of research of myeloid-derived suppressor cells (MDSCs) due to their ability to suppress immune responses. MDSCs immunoregulatory role in transplant has been highlighted in previous studies performed in animal models that led to suggest them as potential biomarkers for promoting allograft tolerance (4, 5). MDSCs were initially described in cancer more than 10 years ago, but their value has been enhanced more recently due to the studies that point their role as important regulators in different clinical settings, such as transplant rejection, infection and autoimmunity (6–10).

In a first report of MDSCs in mice, these cells were described as a CD11b^+^ Gr1^+^ subpopulation and additional experimental models demonstrated their role in the induction of tolerance (5, 11). In renal transplant patients Luan et al. (12) featured MDSCs, as CD33^+^ CD11b^+^ HLA-DR^-^ cells. Those cells were capable of promoting a Treg phenotype *in vitro* and correlated with the percentage of Treg *in vivo*. Meng et al. (13), found that MDSCs were related to increased graft survival and those MDSCs obtained from transplant recipients were also able to expand regulatory T cells (14). Taking together, these studies point out MDSCs as main players involved in tolerance processes. Within the human MDSCs population, three main subsets have been identified: monocytic-MDSCs (Mo-MDSCs: CD33^+^CD11b^+^ HLA-DR^-^ CD14^+^), polymorphonuclear-MDSCs (PMN-MDSCs: CD33^+^CD11b^+^ HLA-DR^-^ CD15^+^), and a population lacking linage surface markers described as early-stage MDSCs (e-MDSCs: CD33^+^HLA-DR^-^CD15^-^ CD14^-^) (15). Because of the current deficiency of specific markers, MDSCs definition needs demonstration of their regulatory function (16). Some studies have shown that MDSCs differentiation and function are affected by existing immunosuppressive drugs (14, 17, 18) but due to the scarce data regarding MDSCs in clinical organ transplantation, further investigations are required to determine their role in graft acceptance and their potential use as biomarkers in order to assess their therapeutic potential in transplantation. Here, we monitored the MDSCs in LTR and assessed their function in association with tacrolimus treatment and also with early clinical events after lung transplantation.

## Materials and Methods

### Study Design

Blood was drawn from 82 patients on the waiting list for lung transplantation in the Hospital Universitario Marqués de Valdecilla since 2016. The study was approved by the Hospital Universitario Marqués de Valdecilla Ethics Committee (CEIC), the patients received the informed consent and agreed to enroll in the study. The mean follow-up time was 239 days. The main immunological and clinical features of the lung transplant recipients (LTR) are summarized in **Table 1**. A protocol biopsy 21 days after lung transplantation was performed in all recipients. Acute rejection was assigned based on ISHLT guidelines (19). Clinical data were collected from patient records and blood was drawn at day 0 (n=82), 7 (n=52), 21 (n=73), 90 (n=67), 180 (n=61) and 360 (n=50) days after transplantation. Importantly, all the LTR were receiving a Tacrolimus-based triple immunotherapy during the first 360 days after transplantation (**Table 1**).

**Table 1:** Main features of study population.

LTP	N = 82
**Age, mean, years**	56.38 (SD 10.34)
**Female**	27 (32.93%)
**PGD**	22 (26.82%)
**Preexisting anti-HLA antibodies**	22 (26.83%)
Class I antibodies	22(26.83%)
Class II antibodies	3 (3.65%)
**Rejection**	30 (36.58%)
**Basal Disease**	
Bronchiectasis/Cystic fibrosis	8 (9.74 %)
In-tur-STISH-ul	44 (53.65 %)
COPD	22 (26.8 %)
PPH	5 (6.09 %)
Others	3 (3.65 %)
**Intubation time**	
≤3 days	66 (80.48 %)
>3 days	16 (19.51 %)
**Infection (first month)**	30 (36.58 %)
**Induction treatment**	
Basiliximab	82 (100%)
**Immunosupressive protocol**	
Calcineurin inhibitor	82 (100 %)
**ABDR Mismatches**	
>3	68 (82.92 %)
≤3	14 (17.08 %)
**Class II Mismatches**	
0	3 (3.66%)
1	35 (42.68%)
2	44 (53.66%)

SD, standard deviation; PGD, primary graft dysfunction; In-tur-STISH-ul diffuse intersticial; COPD, chronic obstructive pulmonary disease; PPH, primary pulmonary hypertension.

### Monoclonal Antibodies and Flow Cytometry Analysis

The following monoclonal antibodies were used to stain peripheral blood mononuclear cells (PBMCs) or isolated MDSCs: anti-CD3-Fluorescein isothiocyanate (FITC) (clone UCHT1), Anti-CD33-Allophycocyanin (APC) (clone D3HL60.251), anti-CD11b-Phycoerytrin (PE)-cyanin 7 (Cy7) (clone Bear1) and anti-CD14-Phycoerytrin-Texas Red- (ECD) (clone RMO52) (Beckman Coulter, Marseille, France); anti-HLA-DR-Brilliant Violet 510 (BV510) (clone L243), anti-CD16-(APC)- Cy7 (clone 3G8) and anti-CD56-FITC (clone HCD56) (Biolegend, San Diego, CA); anti-CD14-FITC (clone MφP9) and anti-CD19-FITC (clone 4G7) (BD Biosciences); anti-CD4-APC-Vio770 (clone REA623) from Miltenyi Biotech (Bergisch Gladbach, Germany) and anti-CD15-Pacific Blue (PB) (clone MCS-1) (Inmunostep, Salamanca, Spain). PBMCs or the cells collected after culture were incubated during 20 min, washed with Phosphate Buffer Saline (PBS) and acquired in a Cytoflex^®^ flow cytometer (Beckman Coulter). Total MDSCs were defined as CD33^+^CD11b^+^HLADR^-^ cells as previously described (14). Further, the gating strategy suggested by Bronte et al. (15) was used to analyze MDSCs subsets by flow cytometry: Mo-MDSCs (CD33^+^CD11b^+^HLADR^-^ CD14^+^ CD15^-^), PMN-MDSCs (CD33^+^CD11b^+^HLADR^-^ CD15^+^ CD14^-^) and e-MDSCs Lin^-^ CD33^+^CD11b^+^HLADR^-^ CD14^-^CD15^-^. Fluorescence Minus One controls were used to assess the HLA DR^+^ and HLA DR^low/-^ populations. The gating strategy is summarized in **Supplementary Figure 1**.

### Cell Isolation and Sorting

To test the suppressive capacity of human MDSCs *in vitro*, human PBMCs were isolated from buffy coats obtained from both Healthy Controls (HC) and LTR (under calcineurin inhibitor treatment during at least 2 years) by Ficoll density gradient centrifugation. To sort CD33^+^ HLA-DR^-^ CD14^+^ cells (Mo-MDSCs), in a first step, the CD33^+^ cells were isolated from PBMCs by magnetic-automated cell sorting (positive selection) Microbeads (Miltenyi Biotech, Bergisch Gladbach, Germany) according to the manufacturer´s instructions. Secondly, Mo-MDSCs were isolated from the CD33^+^ enriched fraction by fluorescence activating cell sorting on a FACS-ARIA II (BD Biosciences, San Jose, CA). The purity of the cell sorting was tested, and > 98% efficiency was accepted for the study.

### *In Vitro* Evaluation of MDSCs Function

CD4^+^ T cells were obtained from HC PBMCs by immunomagnetic isolation using EasySep™ Human CD4+naïve T Cell Isolation Kit (Stemcell Technologies, Grenoble, France) and incubated with CarboxyFuorescein Succinimidyl Ester (CFSE). The CFSE-labeled T CD4^+^ cells (5x10^5^) were stimulated with Dynabeads Human T-activator CD3/CD28 (Life Technologies AS, Oslo, Norway) in U-bottomed 96-well plates (Thermo Fisher Scientific, Hvidovre, Denmark) with complete RPMI media supplemented with 10% human AB+ serum. In order to determine the suppressive function of MDSCs subsets, autologous Mo-MDSCs were added to the culture at 1:2 ratio (CD4^+^T cells:MDSCs) and proliferation was analyzed after 5 days of culture by flow cytometry. These experiments were repeated five times for each donor. The same functional assays were replicated at least four times using blood from different donors and at least two times using blood from LTR.

### Statistical Analysis

To test if the variables followed a Gaussian distribution we performed Kolmogorov Smirnoff test. For those non-parametric unpaired variables, Mann-Whitney U test was used to compare two groups and Kruskal-Wallis (not matching) or Friedman (repeated measures) test were used to compared more than two groups. To check parametric unpaired variables Student´s t test was used to compare two groups and more than two groups were compared using the parametric analysis of variance (ANOVA) as appropriate. Differences between two paired groups were assessed using the Student´s t-test for paired data or the Wilcoxon signed-rank test when data were or not normally distributed, respectively. Multiple comparisons were assessed using Dunn or Tukey´s tests. Statistical analyses were performed using Graphpad software version 8.4.3 (GraphPad Inc. San Diego, CA). To examine the relationship between variables, the Pearson correlation was calculated by using SPSS Statistics version 24.

## Results

### Monitoring MDSCs in Lung Transplant Patients

**Figure 1:**
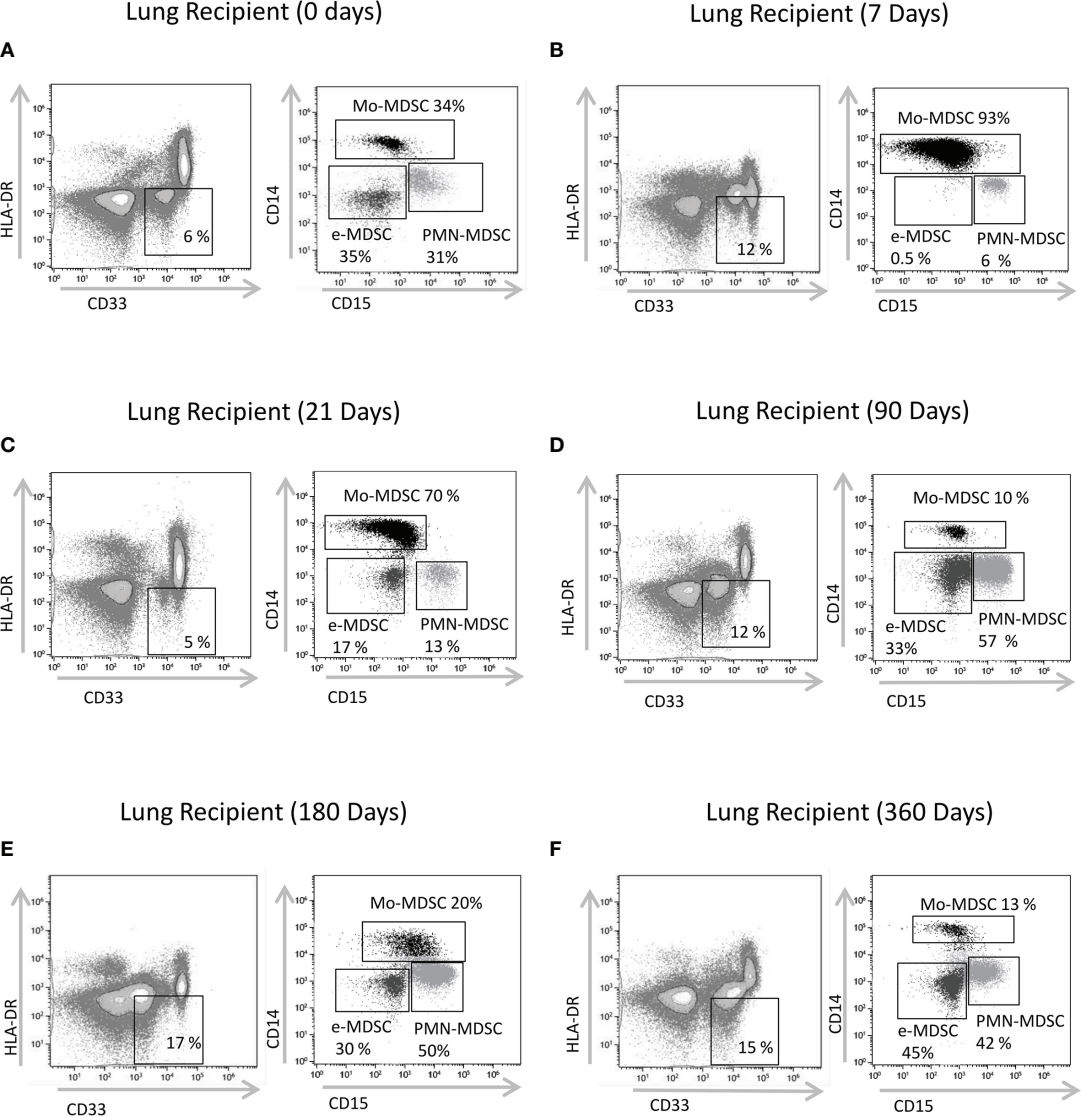
Characterization of MDSCs subsets by flow cytometry. CD33^+^ CD11b^+^HLA-DR^-^ myeloid cells were selected from live cells after doublets and debris exclusion (CD11b expression not shown). To define monocytic (Mo-MDSCs), early-stage (e-MDSCs) and polymorphonuclear (PMN-MDSCs) MDSCs, the CD14 and CD15 expression was analyzed on cells selected from CD33^+^HLA-DR^-^ MDSCs. Representative flow cytometry data of MDSCs from **(A)** patients on the day of transplantation (day 0); lung transplant recipients on days **(B)** 7, **(C)** 21, **(D)** 90, **(E)** 180 and **(F)** 360 post-transplantation is shown. % subsets calculated from total MDSCs.

**Figure 2:**
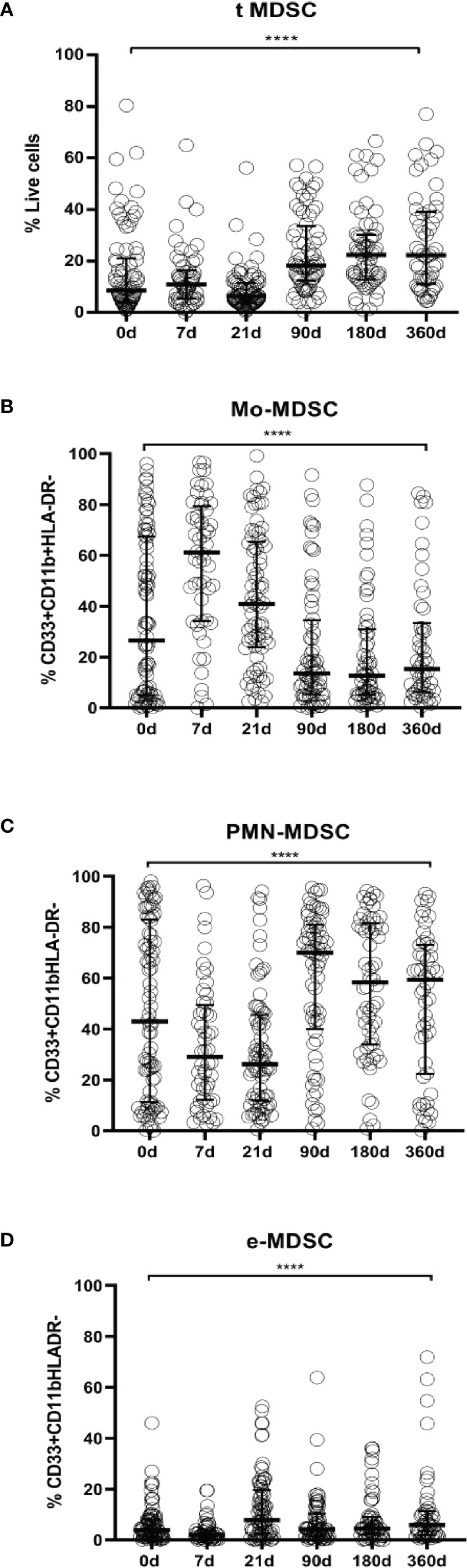
MDSCs frequencies in LTR. **(A)** Frequencies of total myeloid-derived suppressor cells (t-MDSCs) from live peripheral blood mononuclear cells (PBMC); **(B)** monocytic-MDSCs (Mo-MDSCs); **(C)** polymorphonuclear MDSC (PMN-MDSCs) and **(D)** early stage-MDSCs (eMDSCs) are shown. Differences between groups were assessed by Kruskal-Wallis and Mann-Whitney U test (****p < 0.0001). % of subsets was calculated from total MDSC. Blood was drawn at day 0 (n=82), 7 (n=52), 21 (n=73), 90 (n=67), 180 (n=61) and 360 (n=50) post-transplantation.

**Figure 3:**
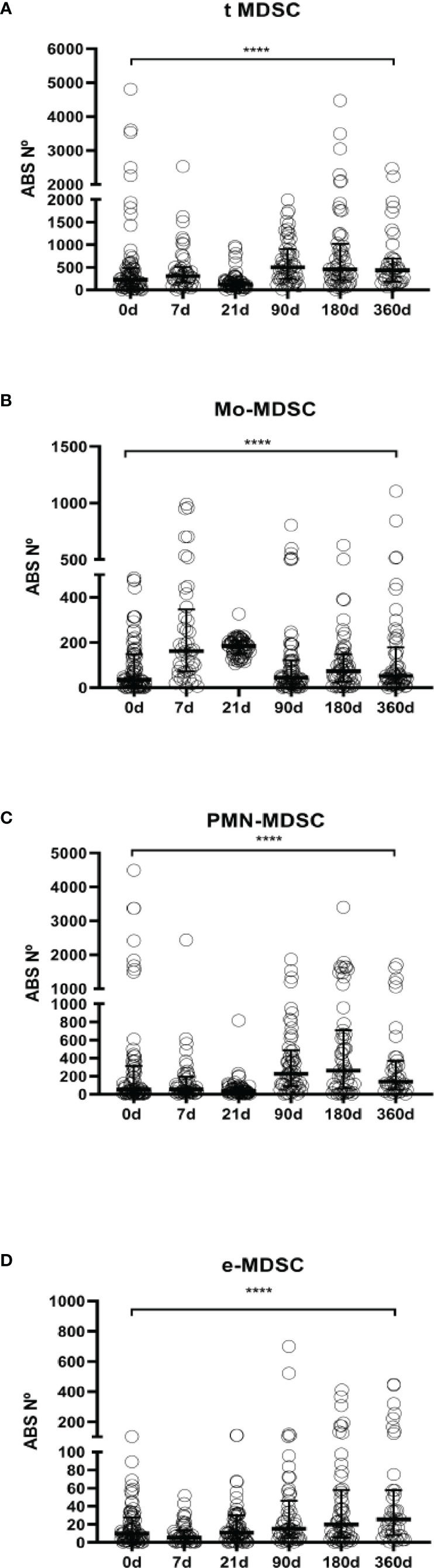
MDSCs absolute numbers in LTR. **(A)** Frequencies of total myeloid-derived suppressor cells (t-MDSCs) in peripheral blood mononuclear cells (PBMCs); **(B)** monocytic-MDSCs (Mo-MDSCs); **(C)** early stage-MDSCs (e-MDSCs) and **(D)** polymorphonuclear MDSCs (PMN-MDSCs) are shown. Differences between groups were assessed by Kruskal-Wallis and Mann-Whitney U test; ****p < 0.0001. Blood was drawn at day 0 (n=76), 7 (n=45), 21 (n=61), 90 (n=55), 180 (n=56) and 360 (n=39) post-transplantation.

Theoretically, MDSCs frequencies might serve as convenient biomarkers to predict clinical outcome after lung transplantation. Therefore, we quantified total MDSCs and MDSCs subsets: Mo-MDSCs, PMN-MDSCs and e-MDSCs in peripheral blood from end-stage lung disease (ESLD) and lung transplant recipients (**Figures 1**–**3**). Paired comparisons of MDSCs subsets frequency in different timepoints are shown in **Supplementary Figures 1**, **2**.

We found that total MDSCs frequencies from ESLD patients and from short-term after transplantation patients remain at baseline levels but they are increased 90 days after transplantation up to a year follow up [ESLD (median 8.49% IQR 4.05%-21.05%) vs 90 days after transplantation (median 18.21%, IQR 12.41%-33.60%) (p=0.0002), ESLD vs 180 days after transplantation (median 22.29, IQR 12.83%-30.21%) (p<0.0001), ESLD vs 360 days after transplantation (median 22.25% IQR 11.06%-39.14%) (p<0.0001)]. (**Figure 2A**, absolute numbers in **Figure 3A**, paired tests in **Supplementary Figures 1A** and **2BA**).

Similarly, we examined changes in MDSCs subsets allocation after transplantation. The evaluation of Mo-MDSCs frequencies revealed that percentages were increased promptly after transplantation and decreased progressively recovering basal levels during the time course follow up [ESLD (median 26.45%, IQR 4.96%-67.41%) vs 7 days post transplantation (median 61.16% IQR 34.12%-79.39%) (p=0.0002), 7days vs 21 days after transplant (median 40.86 IQR 23.73%-65.37%) (p<0.0001), 7days vs 90 days after transplantation (median 13.56% IQR 5.41%-34.47%) (p<0.0001), 7days vs 180 (median 12.63% IQR 5.22%-31.02%) (p<0.0001) and 7days vs 360 days after transplant (median 15.27%, IQR 6.26%-33.39%) (p<0.0001)] (**Figure 2B**, absolute numbers in **Figure 3B**, paired tests in **Supplementary Figures 1B** and **2B**).

On the other hand, PMN-MDSCs frequencies on the short-term after transplantation were significantly lower up to 90 days; then they stayed increased during the time course follow up [7 days after transplantation (median 29.03%; IQR 12.22%-49.03%) vs 90 days (median 69.95% IQR 39.99%-81.04%) (p< 0.0001), 7 days vs 180 days (median 58.28% IQR 33.93%-81.47%) (p=0.0007). 21 days (median 26.19% IQR 11.95%-45.58%) vs 90 days (p<0.0001), 21 vs 180 days (p<0.0001) and 21 vs 360 days (median 59.38% IQR 22.34%-73.13%)(p=0.0184)] (**Figure 2C**, absolute numbers in **Figure 3C**, paired tests in **Supplementary Figures 1C** and **2C**).

The effect of transplantation on e-MDSCs frequencies was calculated as well. We observed e-MDSCs basal levels are low at baseline and post transplant compared to PMN-MDSCs and Mo-MDSCs. Nevertheless, 21 days after transplantation there is an increase (median 7.742%; IQR 2.5%-19.63%) compared to pre transplant levels (median 3.92%; IQR 1.35%-8.06%) (p=0.0375) and 7 (median 2.04%; IQR 0.84%-3.93%) (p<0.0001) (**Figure 2D**, absolute numbers in **Figure 3D**, paired tests in **Supplementary Figures 1D** and **2D**).

No significant differences were found when comparing MDSCs frequencies from ESLD and HC (n=59) matched by sex and age (data not shown).

### MDSCs From Tacrolimus Treated LTR Effectively Suppress T Cell Proliferation *In Vitro*

Because of the lack of specific markers, MDSCs need to be identified upon demonstration of their regulatory function. Then, we determined the cell-suppressive ability of MDSCs from healthy controls and tacrolimus treated LTR. Moreover, as LTR were under Tacrolimus treatment, it is important to evaluate the potential effect of the immunosuppressive treatment on MDSCs.

**Figure 4:**
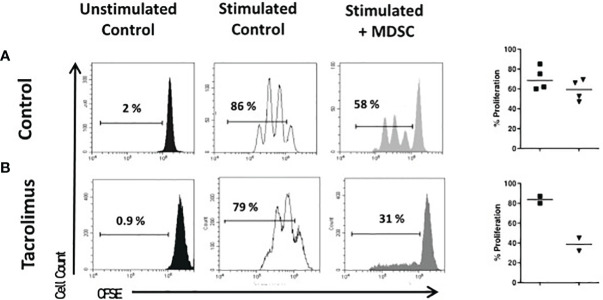
Suppressive function of MDSCs. Sorted CD4 ^+^ T cells were stained with carboxyfluorescein-succinimidyl ester (CFSE) and cultured under polyclonal activation alone or with autologous monocytic-myeloid-derived suppressor cells (Mo-MDSCs). Representative flow cytometry plots of two independent experiments with Mo-MDSCs from healthy volunteers **(A)** and two lung transplant recipients under tacrolimus treatment **(B)** are shown. The summary of % of proliferation stimulated (black squares) and with MDSCs (black triangles) of 4 healthy controls (top panel) and 2 LTR with tacrolimus treatment (bottom panel).

The suppressive capacity of Mo-MDSCs was analyzed using an *in vitro* assay of polyclonally-activated T cell proliferation. Sorted Mo-MDSCs were added at a 1:2 ratio to autologous CD3/CD28-stimulated CD4^+^ T cells. Two patients under long-term tacrolimus treatment and four HC were tested (**Figure 4**). Results indicate that Mo-MDSCs obtained from tacrolimus treated LTR were significantly more suppressive in comparison with HC. This suggests that Mo-MDSCs from transplant patients exhibit potent suppressive function *in vitro* despite of the immunosuppressive treatment.

### MDSCs and Clinical Events

**Figure 5:**
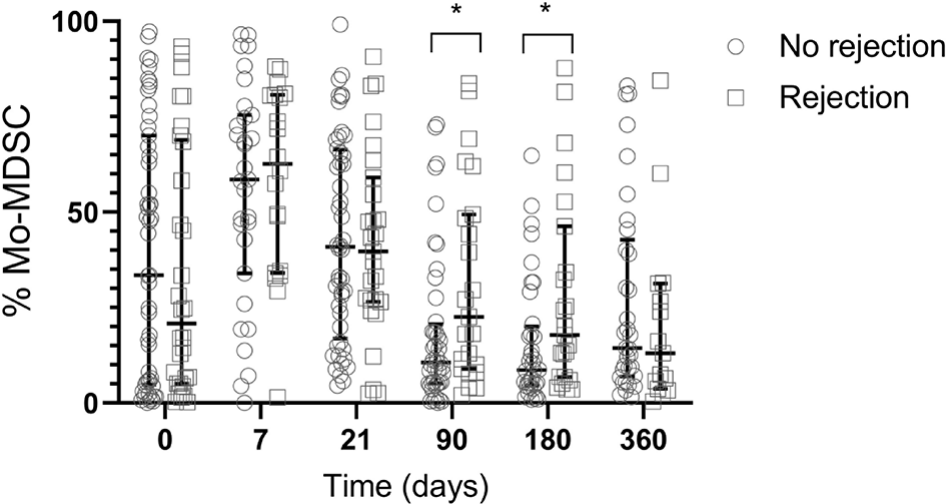
Frequency of Mo-MDSCs and acute rejection. Comparison of median frequency of Mo-MDSCs in lung transplant recipients with rejection (white squares) and no rejection (white circles). At 90 and 180 days post-transplant Mo-MDSCs percentages were lower in patients who do not reject compared to those who reject. Box represents median and 25th and 75th percentiles and whiskers were calculated by the Tukey method; *p < 0.05.

To evaluate if MDSCs can modulate the balance between rejection and graft acceptance, we next examined the effect of MDSCs subsets frequency on clinical events. In our cohort, we found no association between MDSCs levels and acute cellular rejection (ACR). In spite of this, we observed an immediate increased of Mo-MDSC post ACR (90 days posttransplant ACR: n=23, median 22.58 IQR 8.96- 83.74; No ACR: n=44, median 10.63 IQR 5.15-20.63) (p=0.0336) and 180 days post-transplant: ACR: n=23, median 17.8 IQR 6.82- 46.28; No ACR n=36, median 8.6 IQR 4.53-20.02) (p=0.0342) (**Figure 5**). Whether this effect is a consequence of the rejection itself or it is produced by the treatment, remains unknown. We found no differences when we studied MDSCs subsets from patients previously sensitized, primary graft disfunction (PGD), primary disease (**Supplementary Figure 3**), sex or gender. No correlation was observed when we studied tacrolimus levels in peripheral blood and MDSCs frequencies (data not shown).

## Discussion

MDSCs represent a varied group of myeloid regulatory cells known for their role in immune regulation (20, 21). Previously published studies performed in animal models that point to them as potential players in the induction of graft acceptance in transplantation (22, 23). Although some studies about MDSCs in human organ transplantation have been reported (6, 12, 13, 18, 24, 25), this is the first study concerning the monitoring of MDSCs in human LTR. In the current study, MDSCs frequencies in 82 LTR were analyzed at multiple time points over the first year after transplantation. In our cohort, we found that total MDSCs percentages increase 3 months post lung transplant up to a year follow up. When we evaluated the changes on MDSCs subsets, we observed increased percentages promptly after transplantation that decreased gradually during the monitoring. On the other hand, the frequencies of those cells with PMN-MDSCs phenotype decreased in the short-term post transplantation and increased during the follow up, although no changes were observed compared to pre-transplant levels. E-MDSCs were significantly increased after transplant compared to ESLD. Previously, we examined the function and the dynamic changes in the frequencies of MDSCs in a cohort of 38 kidney transplant recipients (KTR) at different time-points. Our previous data indicate that Mo-MDSCs frequencies increase at 6 months and remain increased up to a year of follow up (14). Agreeing with our results, Luan et al. (12) reported that MDSCs frequencies were elevated from 3 to 12 months after transplant. Utrero-Rico et al. (24), described Mo-MDSCs cells counts were increased within the first days after transplant in KTR, in spite of the use of induction and immunosuppressive therapy and these cells counts remained high for one year after transplant.

Hock et al. showed that MDSCs subsets increased promptly after transplant in KTR and then underwent some variations during the first year of follow-up (6). Interestingly, in a different study from the same group, the authors found that most of those patients with long term transplants had increased MDSCs numbers, which suggests that MDSCs expand on the long term (26). Taken together, these reports point towards an increase in the frequency of MDSCs rapidly after transplantation, peaking after immunosuppressive therapy administration. Furthermore, when MDSCs from donors who had undergone parallel surgical methodologies to the recipients were analyzed, the evidence suggested that the changes observed in KTRs were likely due to the immunosuppressive therapy rather than the inflammation caused by the surgery (26). Aligned with these results, the release of neutrophils from the bone marrow secondary to glucocorticoids is well established (27) and the induction of anti-inflammatory monocytes resembling MDSCs (28, 29) in response to glucocorticoids has also been described. In essays performed in the immunomonitoring group in the Hospital Klinikum (HKR) in Regensburg (30) it was observed a reduction in HLA-DR expression in monocytes after dexamethasone exposure. As a consequence, after dexamethasone treatment these monocytes acquired a Mo-MDSCs phenotype. This supports the hypothesis that corticosteroids are increasing Mo-MDSCs population in peripheral blood rapidly after transplantation. In addition, these increases suggest that MDSCs numbers are not negatively affected by the tacrolimus-based maintenance therapy.

In a previous report from our group we evaluated the function of MDSCs obtained from KTR under calcineurin (tacrolimus) or mTOR (rapamycin) inhibition at 360 days of immuno-suppressant maintenance and we observed that MDSCs from tacrolimus, but not rapamycin treated KTR, were able reduce effectively CD4^+^ T cell proliferation *in vitro* (14). Calcineurin inhibitors are immunosuppressive drugs regularly used in transplantation primarily to prevent T cell activation and expansion, hence understanding their effect on MDSCs is critical to develop strategies to promote allograft acceptance in transplantation. In a mouse model of skin transplant, cyclosporine A (CsA) treatment increased the expression of indoleamine 2,3-dioxygenase (IDO) and enhanced the suppressive function of MDSCs in allograft recipients (30). Here, we report that the suppressive capacity of cells with a Mo-MDSCs phenotype obtained from long-term tacrolimus treated LTR is enhanced compared to the suppressive function of MDSCs obtained from healthy donors.

Heigl et al, featured MDSCs in LTR to determine if MDSCs can serve as a potential target in the field (25). They showed functional G-MDSCs obtained from LTR and described a mild correlation with CNI levels, as previously reported (17, 31).

It has been described that FK binging protein (FKBP) is expressed in MDSCs and PMN-MDSCs from tumoral animal models and modulates their suppressive function (32). Altogether, studies on the functional activity and number of MDSCs in transplantation, suggest that immunosuppressive treatments such as glucocorticoids and CNI are able to modulate mobilization and function of MDSCs.

Mycophenolic acid (MPA) is a non-competitive inhibitor of inosine monophosphate dehydrogenase widely used in immunosuppression regimens. MPA avoids the conversion of inosine monophosphate to guanine monophosphate. Thus, by blocking the *de novo* synthesis of purines, MPA acts as a potent inhibitor of the proliferation of lymphocytes (33). Among its anti-inflammatory effects, the effects of MPA on monocyte- macrophage lineage cells have been described (34): decreased recruitment of monocytic lineage cells into sites of graft rejection, decreased production of IL-1B and increased production of the IL-1 receptor antagonist (35). However, although mycophenolic acid is likely to be involved in MDSCs development, this effect has not been explored in this report. Also, other limitation of the present study is that our cohort of LTR was under the same immunosuppressive treatment, and differences in the effect of immunosuppressive drugs can not be assessed to stablish to what extend the drugs are modulating MDSCs effects differentially.

As MDSCs frequencies could serve as biomarkers to predict clinical outcome after lung transplantation, we collected data regarding the presence of some clinical events and we examined those clinical outcomes in relation to MDSCs frequencies. In contrast to some studies (13, 24), we describe here that the frequencies of Mo-MDSCs 90 and 180 days post-transplant are higher in patients that suffered acute cellular rejection (ACR) of the graft compared to those who did not.

Similarly to previous results, when Mo-MDSCs were treated with dexamethasone (30), Okano et al. (18) reported that MDSCs numbers increased to 6 times in an intestinal transplant patient on day 3 after methylprednisolone treatment for ACR. Hence, these post transplant changes in MDSCs frequency related to clinical outcomes might be reflecting changes in glucocorticoid treatment. Interestingly, in a long-term retrospective study, in those patients with more than 10-year standing kidney grafts and low immunosuppression, Mo-MDSCs were significantly higher than in short-term renal recipients, and Mo-MDSCs levels correlated with survival rates (36, 37). According to previous mentioned studies that point out MDSCs are regulated by immunosuppressive treatments such as CNI, we also found slightly higher levels of tacrolimus in peripheral blood of patients with rejection, suggesting that MDSCs frequencies could also be modulated by the tacrolimus-based maintenance therapy.

In summary, the results of this study demonstrate that mobilization of MDSCs subsets is differentially regulated by yet undetermined stimulus, but immunosuppressive therapy is likely involved in the modulation of MDSCs numbers and function after transplant.

Also, the increases in Mo-MDSCs after acute rejection raise the question whether MDSCs could be implicated in the development of chronic lung allograft dysfunction (CLAD). This study confirms the ability of Mo-MDSCs subsets from tacrolimus treated LTR to suppress T cell proliferation and raise the possibility that MDSCs may play an important role in suppressing allogeneic immune responses.

## Data Availability Statement

The raw data supporting the conclusions of this article will be made available by the authors, without undue reservation.

## Ethics Statement 

The studies involving human participants were reviewed and approved by 2019.222 (PI19/01509) the Hospital Universitario Marqués de Valdecilla Ethics Committee (Santander, Spain). The patients/participants provided their written informed consent to participate in this study.

## Author Contributions

MI-E: data acquisition, analysis and interpretation, investigation, methodology, writing, and original draft. DS: conceptualization,formal analysis, funding acquisition, supervision, writing, review, editing, and final approval of the version to be published. DM-F: data acquisition, analysis and interpretation, investigation, and methodology. VMM-C: investigation, methodology (provided and cared for study patients), and clinical data interpretation. PL: investigation and methodology. MA-P: investigation and methodology. SR: investigation and methodology. DI-F: investigation, methodology (provided and cared for study patients), and clinical data interpretation. SR-F: investigation, methodology (provided and cared for study patients), and clinical data interpretation. JC: investigation, methodology (provided and cared for study patients), and clinical data interpretation. ML-H: conceptualization, funding acquisition, formal analysis, supervision, writing, review, editing, and final approval of the version to be published. All authors contributed to the article and approved the submitted version.

## Funding

This work was supported by grants from the FIS-ISCII (PI16/01585) to ML-H and NVAL16/22 to DS.

## Conflict of Interest

The authors declare that the research was conducted in the absence of any commercial or financial relationships that could be construed as a potential conflict of interest.

## Publisher’s Note

All claims expressed in this article are solely those of the authors and do not necessarily represent those of their affiliated organizations, or those of the publisher, the editors and the reviewers. Any product that may be evaluated in this article, or claim that may be made by its manufacturer, is not guaranteed or endorsed by the publisher.

## Supplementary Material

Supplementary Figure 1 Paired comparison of MDSCs subsets. Paired analysis of total-Myeloid derived Suppressor cells (t-MDSCs) **(A)**, Monocytic (Mo)-MDSCs **(B)**, Poly morpho nuclear (PMN)-MDSCs **(C)**, and early stage (e)-MDSCs **(D)** at day 0 and 7 days after transplant (left panels); at day 7 and day 21 (center-left panels); at day 21 and 90 (center panels); at day 90 and 180 (center-right panels) and at day 180 and 360 days (right panels). Mean values of each MDSC subset are showed in each timepoint. The central number is the difference (in percent) between the means of the two time points. Differences between time points were calculated using the following formula: (mean posTx - mean previous timepoint)/mean previous timepoint. Paired t-test was performed, and p value depicted in each panel.

Supplementary Figure 2 Paired comparisons of MDSC subset frequency changes to pre transplant levels: Paired analysis of total-Myeloid derived Suppressor cells (t-MDSCs) **(A)**, Monocytic (Mo)-MDSCs **(B)**, polymorphonuclear (PMN)-MDSCs **(C)**, and early €-MDSCs **(D)** at day 0 and 7 days after transplant (left panels); at day 0 and day 21 (center-left panels); at day 0 and 90 (center panels); at day 0 and 180 (center-right panels) and at day 0 and 360 days (right panels). Mean values of each MDSCs subset are showed in each timepoint. The central number is the difference (in percent) between the means of the two time points. Differences between time points were calculated using the following formula: (mean posTx-mean preTx)/mean preTx. Paired t-test was performed, and p value depicted in each panel.

Supplementary Figure 3 MDSCs frequencies in LTR and clinical events Frequencies of total myeloid-derived suppressor cells (t-MDSCs), monocytic (Mo)-MDSCs, polymorphonuclear (PMN)-MDSCs, early stage (e)-MDSCs in PBMC were studied and compared with clinical events such as **(A)** rejection **(B)** primary graft dysfunction (PGD), **(C)** basal disease, **(D)** anti-HLA antibodies development. Differences between groups were assessed by Kruskal- Wallis and Mann-Whitney U test (*p<0.05).

## Abbreviations

7AAD, 7-amino-actinomycin D; CNI, Calcineurin Inhibitors; e-MDSC, early stage MDSCs; HC, healthy controls; LTR, Lung Transplant Recipients; Mo-MDSCs, Monocytic MDSCs; MDSCs, Myeloid Derived Suppressor Cells; PBMC, Peripheral Blood Mononuclear Cells; PBS, Phosphate Buffer Saline; PMN-MDSCs, Polymorphonuclear MDSCs.
