# Supplementary figures and images for: Myeloid-Derived Suppressor Cells Are Increased in Lung Transplant Recipients and Regulated by Immunosuppressive Therapy
Source: Front Immunol. 2022 Jan 10;12:788851. doi: 10.3389/fimmu.2021.788851 (PMC8848105; doi:10.3389/fimmu.2021.788851)

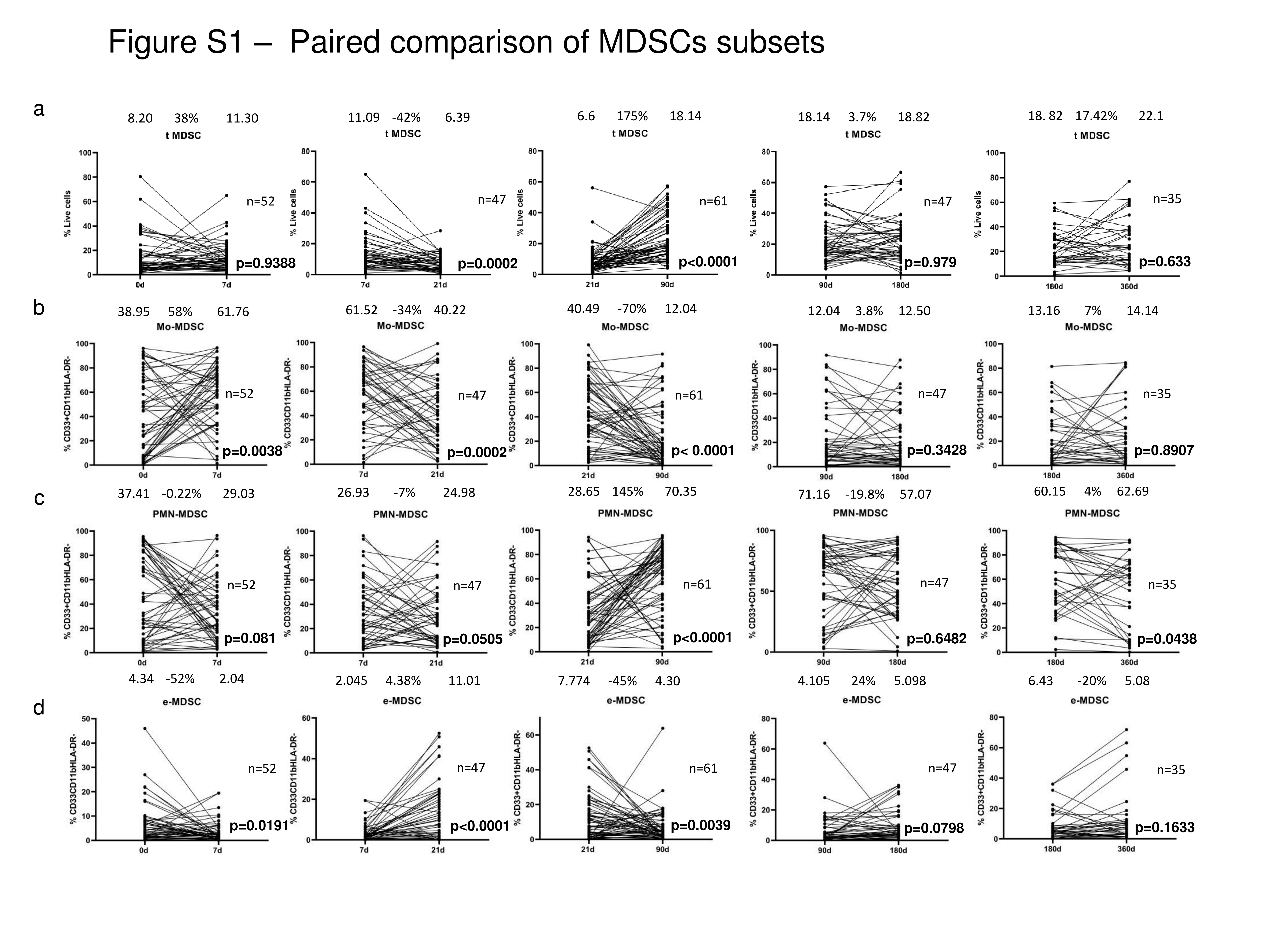

Supplement: Supplementary Figure 1 — Paired comparison of MDSCs subsets. Paired analysis of total-Myeloid derived Suppressor cells (t-MDSCs) (A), Monocytic (Mo)-MDSCs (B), Poly morpho nuclear (PMN)-MDSCs (C), and early stage (e)-MDSCs (D) at day 0 and 7 days after transplant (left panels); at day 7 and day 21 (center-left panels); at day 21 and 90 (center panels); at day 90 and 180 (center-right panels) and at day 180 and 360 days (right panels). Mean values of each MDSC subset are showed in each timepoint. The central number is the difference (in percent) between the means of the two time points. Differences between time points were calculated using the following formula: (mean posTx - mean previous timepoint)/mean previous timepoint. Paired t-test was performed, and p value depicted in each panel. [file Image_1.tif]

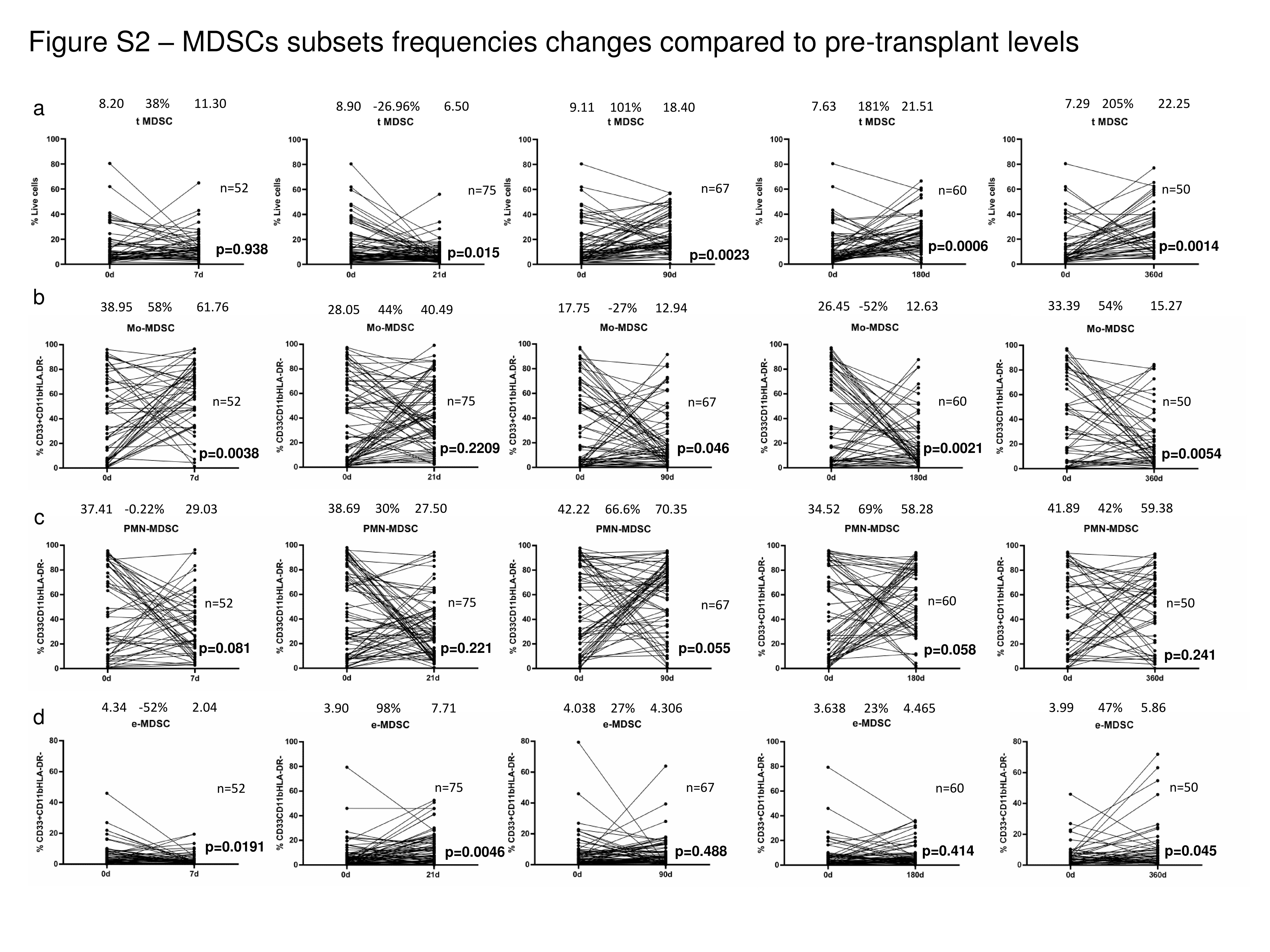

Supplement: Supplementary Figure 2 — Paired comparisons of MDSC subset frequency changes to pre transplant levels: Paired analysis of total-Myeloid derived Suppressor cells (t-MDSCs) (A), Monocytic (Mo)-MDSCs (B), polymorphonuclear (PMN)-MDSCs (C), and early €-MDSCs (D) at day 0 and 7 days after transplant (left panels); at day 0 and day 21 (center-left panels); at day 0 and 90 (center panels); at day 0 and 180 (center-right panels) and at day 0 and 360 days (right panels). Mean values of each MDSCs subset are showed in each timepoint. The central number is the difference (in percent) between the means of the two time points. Differences between time points were calculated using the following formula: (mean posTx-mean preTx)/mean preTx. Paired t-test was performed, and p value depicted in each panel. [file Image_2.tif]

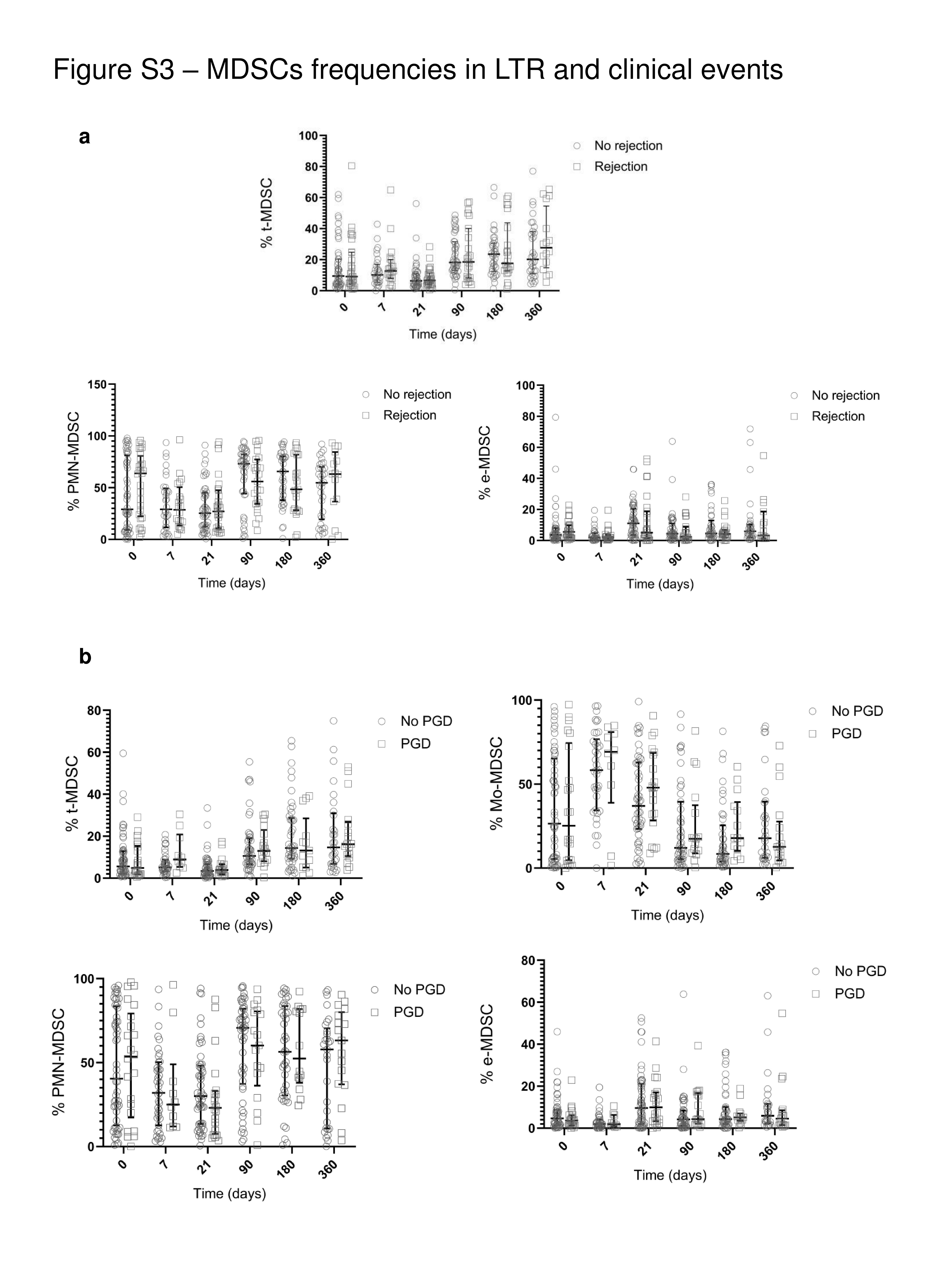

Supplement: Supplementary Figure 3 — MDSCs frequencies in LTR and clinical events Frequencies of total myeloid-derived suppressor cells (t-MDSCs), monocytic (Mo)-MDSCs, polymorphonuclear (PMN)-MDSCs, early stage (e)-MDSCs in PBMC were studied and compared with clinical events such as (A) rejection (B) primary graft dysfunction (PGD), (C) basal disease, (D) anti-HLA antibodies development. Differences between groups were assessed by Kruskal- Wallis and Mann-Whitney U test (*p<0.05). [file Image_3.tif]

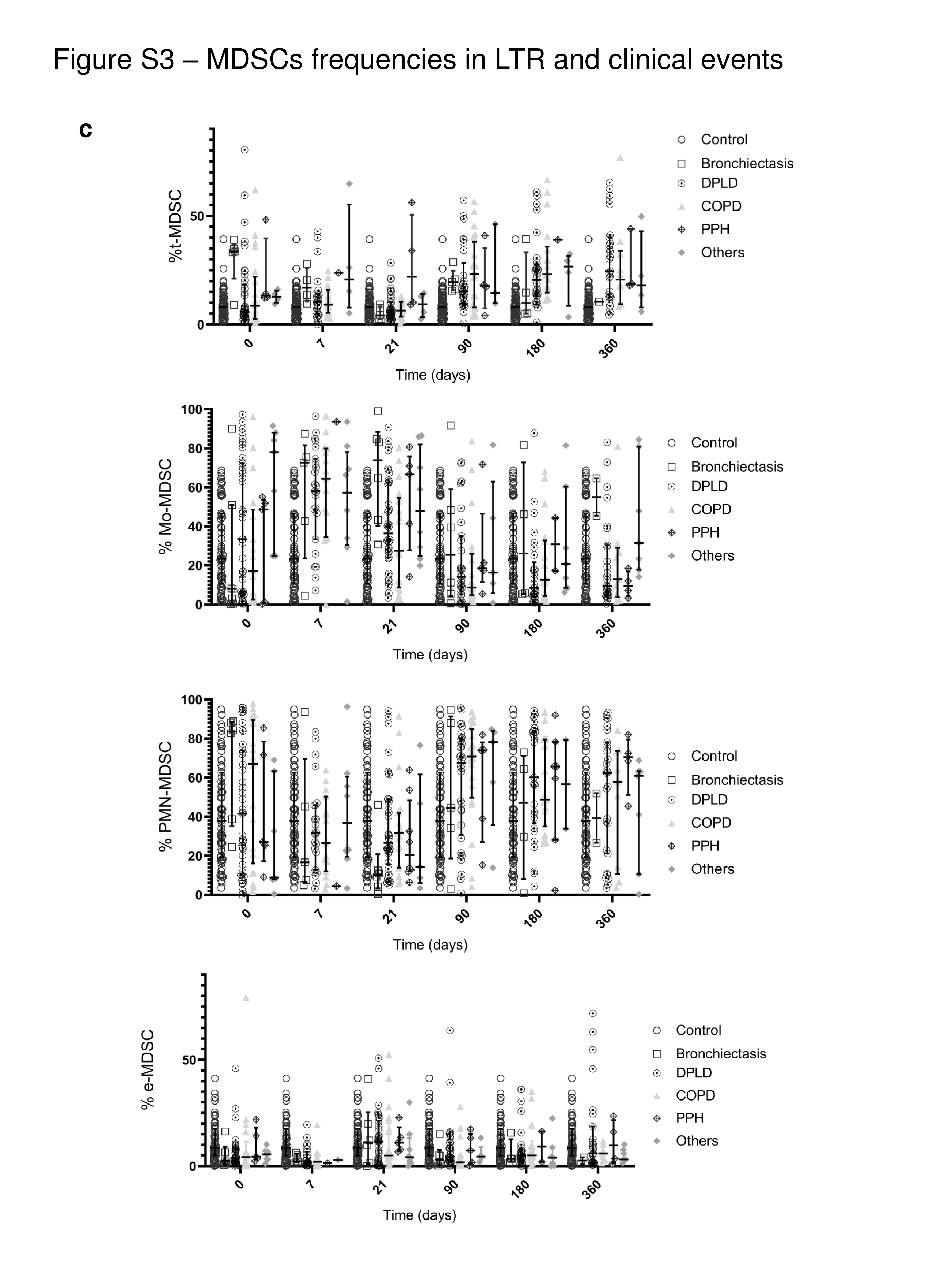

Supplement: Supplementary file 4 [file Image_4.tif]

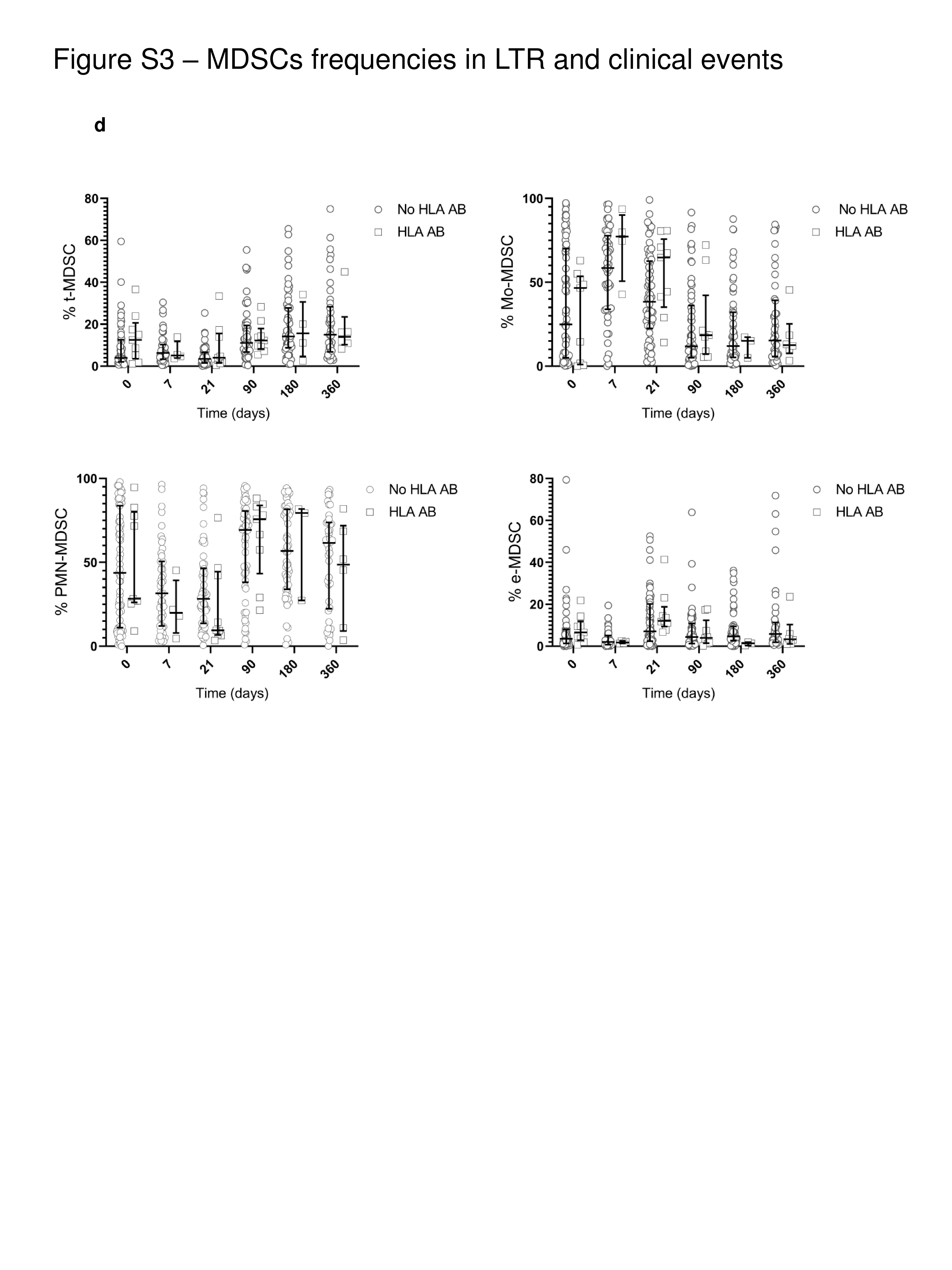

Supplement: Supplementary file 5 [file Image_5.tif]
